# Novel Thiadiazole-Based Molecules as Promising Inhibitors of Black Fungi and Pathogenic Bacteria: In Vitro Antimicrobial Evaluation and Molecular Docking Studies

**DOI:** 10.3390/molecules27113613

**Published:** 2022-06-04

**Authors:** Huda R. M. Rashdan, Mohamad T. Abdelrahman, Ihsan A. Shehadi, Sara S. El-Tanany, Bahaa A. Hemdan

**Affiliations:** 1 Chemistry of Natural and Microbial Products Department, Pharmaceutical and Drug Industries Research Institute, National Research Centre, Dokki, Cairo 12622, Egypt; 2 Radioisotopes Department, Nuclear Research Centre, Egyptian Atomic Energy Authority, Cairo 13759, Egypt; medo2medo@gmail.com; 3 Department of Chemistry, Pure and Applied Chemistry Research Group, College of Sciences, University of Sharjah, Sharjah P.O. Box 27272, United Arab Emirates; ishehadi@sharjah.ac.ae; 4 Environmental and Occupational Medicine Department, National Research Centre, Cairo 12622, Egypt; sara_eltanany87@yahoo.com; 5 Water Pollution Research Department, Environmental and Climate Change Research Institute, National Research Centre, 33 El-Bohouth St., Dokki, Giza 12622, Egypt; bahaa_nrc@yahoo.com

**Keywords:** COVID-19 pandemic, 1,3,4-thiadiazoles, hydrazonoyl halides, antimicrobial activity, black fungus, pathogenic bacteria, molecular docking

## Abstract

Novel 1,3,4-thiadiazole derivatives were synthesized through the reaction of methyl 2-(4-hydroxy-3-methoxybenzylidene) hydrazine-1-carbodithioate and the appropriate hydrazonoyl halides in the presence of a few drops of diisopropylethylamine. The chemical structure of the newly fabricated compounds was inferred from their microanalytical and spectral data. With the increase in microbial diseases, fungi remain a devastating threat to human health because of the resistance of microorganisms to antifungal drugs. COVID-19-associated pulmonary aspergillosis (CAPA) and COVID-19-associated mucormycosis (CAM) have higher mortality rates in many populations. The present study aimed to find new antifungal agents using the disc diffusion method, and minimal inhibitory concentration (MIC) values were estimated by the microdilution assay. An in vitro experiment of six synthesized chemical compounds exhibited antifungal activity against *Rhizopus* *oryzae*; compounds with an imidazole moiety, such as the compound 7, were documented to have energetic antibacterial, antifungal properties. As a result of these findings, this research suggests that the synthesized compounds could be an excellent choice for controlling black fungus diseases. Furthermore, a molecular docking study was achieved on the synthesized compounds, of which compounds **2**, **6,** and **7** showed the best interactions with the selected protein targets.

## 1. Introduction

Coronavirus disease 2019 (COVID-19) is a severe public health issue plaguing the world. Due to this, it was announced as a pandemic by the World Health Organization (WHO) within a limited timeframe [1]. Other microbial illnesses were observed in COVID-19 individuals [2]. Severe fungal infections such as aspergillosis caused by *Aspergillus*, *Candida*, mucoromycetes, and others were also identified in COVID-19 sufferers [3,4]. Mucormycosis, or black fungus, is a fungal ailment primarily caused by Mucorales species including *Rhizopus* and *Mucor* [5,6]. As a result, COVID-19-connected mucormycosis (CAM) has been reported in people who have been medicated or recovered [4]. CAM occurrences are on the rise worldwide, especially in Asiatic regions, where COVID-19 sufferers have a high mortality rate [4,7].

The discovery of bioactive components that engage with antibiotic resistance is among the essential issues for exploratory antimicrobial investigation. Pathogenic microorganisms’ resistance to currently available antibiotics is increasingly becoming a significant international hazard. Antibacterial drugs, for example, are known to have no specific effectiveness, considering the molecular similarities between human cells and pathogen types [8].

The dissemination and modifications of horizontal chromosome transfer are among the mechanisms associated with resistant bacteria’s emergence [9]. Resistance to antibiotics evolves and expands due to massive antibiotic intake, trafficking, and multidrug-resistant organisms that do not respond to treatment [10]. Different strains of bacteria have already been found to have fundamental features that promote them to withstand and prevent antibiotic assaults [11]. Separated *Staphylococcus aureus* isolates, for example, have already shown compositional tolerance to a multitude of antibiotic classes, comprising beta-lactams, glycopeptides, aminoglycosides, and fluoroquinolones [12]. Furthermore, *Pseudomonas aeruginosa* is notable for its capacity to survive in highly antibiotic-resistant biofilm communities [13]. Consequently, numerous researches should be undertaken to produce new antibacterial medications with completely distinct chemical components and unique practical benefits [14]. 

The N atom has a unique nature that gives flexibility to the biological targets. Accordingly, *N*-heterocyclic compounds have many therapeutic uses as scaffolds for new drug candidates [15,16,17,18,19,20,21,22,23,24,25,26,27,28,29,30]. In the medicinal chemistry, pyrazine, pyrazoline, indazole, and thiazole derivatives, for example, could offer a broad spectrum of therapeutic and pharmacological applications [31]. 1,3,4-thiadiazoles are of great interest due to their significant importance for investigating potent bioactive compounds with a wide range of applications in the pharmaceutical industry [15,16,32,33,34,35,36,37]

Moreover, molecular docking is a method used to predict the favorable positioning of ligands to a target when bound to each other to form a stable complex [38]. By understanding the energy’s preferred orientation, the strength of the binding affinity between the ligand and target site, and the type of interactions between the ligand and receptor, molecular docking can serve as a first choice procedure for a pharmaceutical drug discovery process [39].

## 2. Results and Discussion

### 2.1. Chemistry

**Scheme 1:**
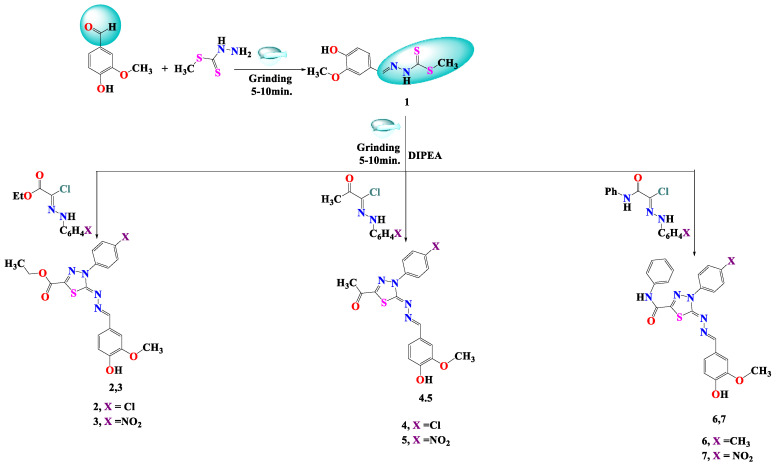
Synthetic procedures of 1,3,4-thiadiazole molecules **2**–**7**.

Methyl-2-(4-hydroxy-3-methoxybenzylidene)hydrazine-1-carbodithioate (**1**) was submitted to react with a set of selected hydrazonoyl halide derivatives utilizing the grinding method at room temperature under the solvent free condition with the addition of catalytic amounts of diisopropyl ethyl amine from two to three drops, to give the desired products **2**–**7**, as illustrated briefly in Scheme 1.

The chemical composition of the newly produced target molecules was affirmed by spectral and microanalytical data as disused briefly in the experimental section.

### 2.2. Antimicrobial Activities

In the current practical research, the in vitro zone of inhibition (ZOI) method was applied to appraise the antibacterial action of six newly synthesized compounds (**2**, **3**, **4**, **5**, **6**, and **7**) against four pathogenic bacteria, namely: *K. pneumoniae*, *P. aeruginosa*, *S. aureus*, and *B. subtilis* using a disc diffusion assay. The potential antibacterial action towards tested pathogenic microbes was expressed as a diameter of the ZOI. Generally, the obtained results exhibited suitable antimicrobial activities for all compounds (Table 1).

The results presented in Table 1 indicate that the highest antibacterial effect was found for compound **7** against all targeted bacterial species, where the diameters of the formed ZOI of the compound **7** against *K. pneumoniae*, *P. aeruginosa*, *S. aureus*, and *B. subtilis* were 22 ± 0.16, 25 ± 0.14, 18 ± 0.28, and 20 ± 0.43 mm, respectively. 

On the other hand, the lowest bactericidal action was observed for compound **3**; the widths of the ZOI were 13 ± 0.23, 15 ±0.36, 10 ± 0.18, and 12 ± 0.29 mm, respectively, for *K. pneumoniae,* *P. aeruginosa*, *S. aureus,* and *B. subtilis*. However, some compounds showed a significant ZOI for bacterial strains: compound **2** (19 ± 0.10, 20 ± 0.23, 13 ± 0.18, and 15 ± 0.25 mm), compound **4** (16 ± 0.18, 18 ±0.28, 13 ± 0.25, and 15 ± 0.42 mm), compound **5** (15 ± 0.35, 17 ±0.24 m 12 ± 0.30, and 14 ± 0.18 mm), and compound **6** (20 ± 0.18, 22 ± 0.23, 15 ± 0.26 m, and 17 ± 0.16 mm), against *K. pneumoniae*, *P. aeruginosa*, *S. aureus,* and *B. subtilis,* respectively. Among these synthesized compounds, **2**, **6,** and **7** were observed to own substantial bactericidal action compared to the reference drugs (ciprofloxacin).

Ciprofloxacin, which is considered one of the most popular antibiotics, was reported to be beneficial against various types of Gram-positive and Gram-negative species [40]. Likewise, it has potent antibacterial properties against *E. coli*, *P. aeruginosa*, *S. aureus*, and *Enterococcus faecium* [41].

Regarding the antifungal activities of the studied compounds, the obtained results indicate that the compounds displayed significantly higher potency for inhibiting the tested black fungal strains, *R. oryzae*, at a concentration of 20 mg/mL for each tested compound, with the ZOI being 9.7 ± 0.11, 10 ± 0.14, 12 ± 0.10, 11 ± 0.14, 14 ± 0.28, and 17 ± 0.14 mm for the synthesized compounds **2**, **3**, **4**, **5**, **6**, and **7**, respectively. 

According to this study, the results obtained display superior antifungal action, and azole drugs had greater efficacy than amphotericin B towards *Aspergillus* species. On the other hand, Azole medicines are believed to be inefficient towards *R. oryzae* [42].

### 2.3. Determination of Minimum Inhibitory Concentration (MIC) and Minimum Bactericidal Concentration (MBC) Values

To further assess the antibacterial potential of the newly synthesized compounds, each compound’s MIC and MBC values were estimated. A series of concentrations (50–300 µg/mL) of six compounds were used to estimate the MIC values and confirm their antibacterial potentials. 

The results of the MIC test exhibited that the potent antibacterial effect and MIC values of compound **7** were 75 µg/mL for *K. pneumoniae* and *B. subtilis*, 100 µg/mL for *P. aeruginosa*, and 125 µg/mL for *S. aureus* (Table 2). Conversely, the lowest antibacterial effect was observed for compound **3,** and MIC values were 275 µg/mL for *K. pneumoniae* and *B. subtilis*, 250 µg/mL for *P. aeruginosa*, and 300 µg/mL for *S. aureus*.

Different concentrations of the newly tested compound were then subjected to the MIC test to confirm their antifungal activities further. Interestingly, as shown in Table 2, the entire tested compound revealed antifungal effects against *R. oryzae*, with different values of MICs. The MIC values were 225, >300, 275, 275, 225, and 150 µg/mL, respectively, for compounds **2**, **3**. **4**, **5**, **6,** and **7** against *R. oryzae*. Results revealed that compound **7** showed higher antibacterial and antifungal activities than all studied compounds.

The biological properties of the thiadiazole ring are multifarious due to it having a wide range of pharmacological actions in which 1,3,4-thiadiazole derivatives are one of the most studied thiadiazole isomers, although 1,3,4-triazole derivatives have few therapeutic measures currently in use during medical practice (e.g., antibacterial activity and carbonic anhydrase inhibitory activity). In contrast, its antibacterial activity has been investigated, and other antimicrobial properties, such as antifungal and antituberculosis abilities, may also be explored [43]. Former investigations have discovered that thiadiazole compounds have a variety of medicinal properties, including analgesic, antimicrobial, antitumor, anticonvulsant, and anti-hepatitis B activity [25,44]. 

Serbanl et al. [9] examined in vitro the antibacterial activity of thiadiazole against *S. aureus*, *Bacillus subtilis*, *E. coli*, and *P. aeruginosa* and its antifungal activity against *R. oryzae* and *Candida albicans* by the disc diffusion technique. The results displayed that decisive antimicrobial action was observed. This phenomenon is attributed to the presence of a sulfur ring in the chemical structure of thiadiazole, which can easily penetrate the cell wall of microbes. The obtained results are in agreement with Er et al. [45] who established that imidazo [2,1-*b*][1,3,4]thiadiazole derivatives displayed significantly forceful antibacterial activities with MIC values of 0.03, 0.03, and 0.5 μg/mL against *S aureus*, *B. subtilis*, and *E. coli*, respectively.

### 2.4. Determination of Extracellular Adenosine Triphosphate (ATP) Level

**Figure 1:**
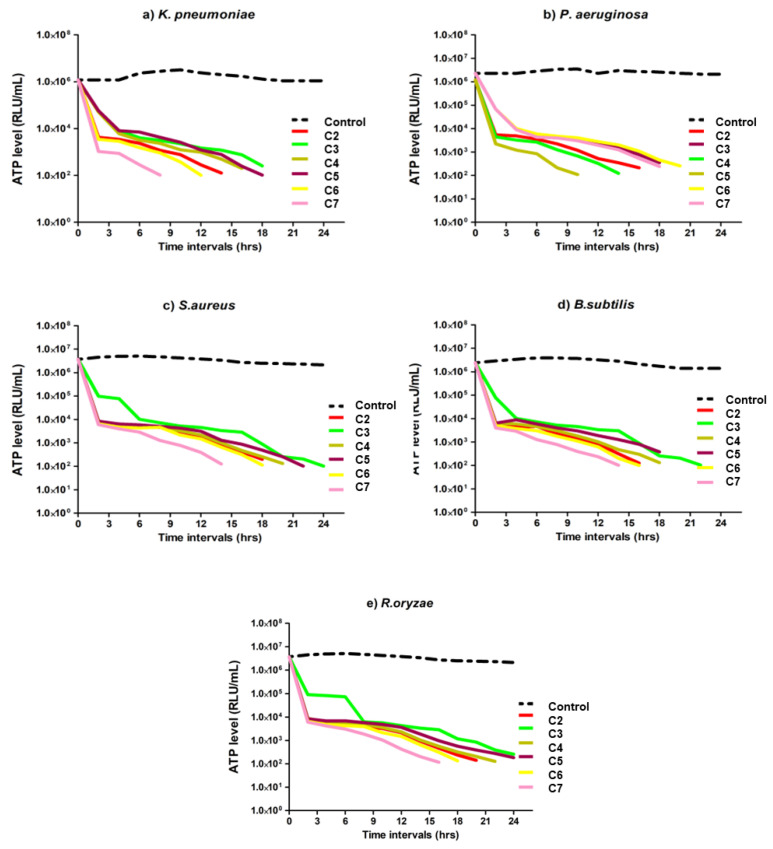
ATP level for (**a**) *K. pneumoniae*, (**b**) *P. aeruginosa*, (**c**) *B. subtilis*, (**d**) *S. aureus*, (**e**), *R. oryzae* before and after exposure to tested compounds.

The total levels of ATP-tested microbes before and after exposure to the effective dose of studied compounds were determined and are linearly presented in Figure 1. In PBS, the initial ATP levels produced for *K. pneumoniae*, *P. aeruginosa*, *S. aureus*, *B. subtilis*, and *R. oryzae* approximately logged 6 RLU/mL. The total ATP levels decreased rapidly in Gram-negative bacteria and then slowly fell in fungal species throughout the experiment, while for the control vials, the total ATP levels remained relatively constant. The results exhibit that the effective dose of the compound **7** showed higher biocidal effects for all tested microbes, where the ATP level disappeared after 10 h for *K. pneumoniae*, after 12 h for *P. aeruginosa*, after 16 h for *B. subtilis*, and after 18 h for *S. aureus* and *R. oryzae* (Figure 1a–e). However, effective concentrations of compound **3** did not affect the fungal strains, indicating that the surfactant did not affect the cellular ATP levels. Similar trends for ATP levels for *S. aureus* were also observed (Figure 1c).

This research exhibits that exposure to the studied compounds resulted in a considerable reduction in cellular ATP levels, symbolizing the banning of bacterial growth due to cell destruction. Typically, the consequences in Figure 1 show that the level of ATP that was significantly decreased in the bacterium *K. pneumoniae* corresponded to the other species tested. In contrast, the decrease level was lower in *R. oryzae*. Similarly, ATP amounts are an exceptional pointer to microbial activity and vibrancy and their capability to multiply and lead the severe infection. Subjecting Gram-negative bacteria to some antimicrobial compounds may diminish the cellular ATP levels and increase cellular protein levels in the culture, proposing a failure of cell membrane integrity [46].

ATP is an essential biomolecule in microbial species because it functions as a versatile energy source. ATP is considered to reveal the existence of live cells of microbes in the environment. On the other hand, as the intracellular level of ATP was indicated to differ with altering environmental and physiological circumstances, it might be utilized as a proxy to point to the microbial metabolic activity in microorganisms [47]. Interestingly extracellular ATP was exploited as the primary source of phosphorus for bacterial growth. In contrast, carbon from ATP molecules was not utilized, implying a prospective function of ATP in biological constancy. Indeed, in cells losing viability, the ability to synthesize ATP is misplaced, and many biochemical reactions, including the action of ATPases, speedily reduce any residual ATP from the cytoplasm [48].

Likewise, the liberation of ATP has to be entertained when considering metabolism or viability, as a considerable portion of the ATP in a culture can be extracellular, particularly in the exponential growth stage or when exposed to antimicrobial agents [49]. Accordingly, ATP values have to be handled with respect, notably as their usage is still argued for some applications. Definitely, the understanding of ATP measurements, especially regarding viability and activity, can be sweetened if the other adenylates (ADP and AMP) are taken into account [50].

### 2.5. Toxicological Performance Assay

**Figure 2:**
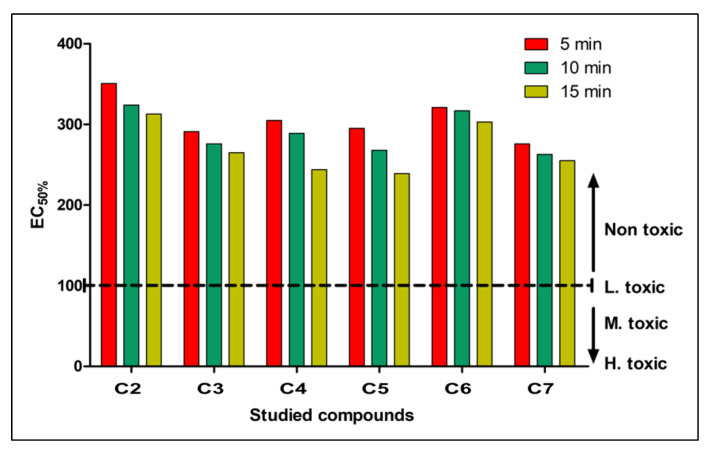
Estimated EC_50%_ level at three different time intervals of all studied compounds.

The conceivable toxicological effects of all studied compounds were successfully measured against the transgenic bacterial cells of the marine bacterium *Aliivibrio fischeri*. As illustrated in Figure 2, the calculated values of EC_50%_ are documented and distinguished for all tested compounds. The toxicological result and effective concentration (EC_50%_) level were registered at three time intervals (5, 10, and 15 min). The results displayed that all tested compounds were secure, lacking toxic effects, and safe for living organisms. All the recorded EC_50%_ readings after 5, 10, and 15 min were less than 100, indicating that these studied compounds were biocompatible and non-toxic (Figure 2). Toxicity testing of wastes, water streams, contaminated sites, and compounds is critical to avoid harmful effects on living organisms and pollution of the environment [51].

Furthermore, the MMT experiment was used to determine the cytotoxic effect of the test compounds by calculating the CC50 and safe dose against HEp-2 cell lines; the obtained findings revealed that the CC50 was 825, 811, 835, 843, 801, and 836 µg/mL, respectively, for C2, C3, C4, C5, C6, and C7, indicating that the synthesized compound had no harmful impact on HEp-2 cells.

## 3. Molecular Docking

**Figure 3:**
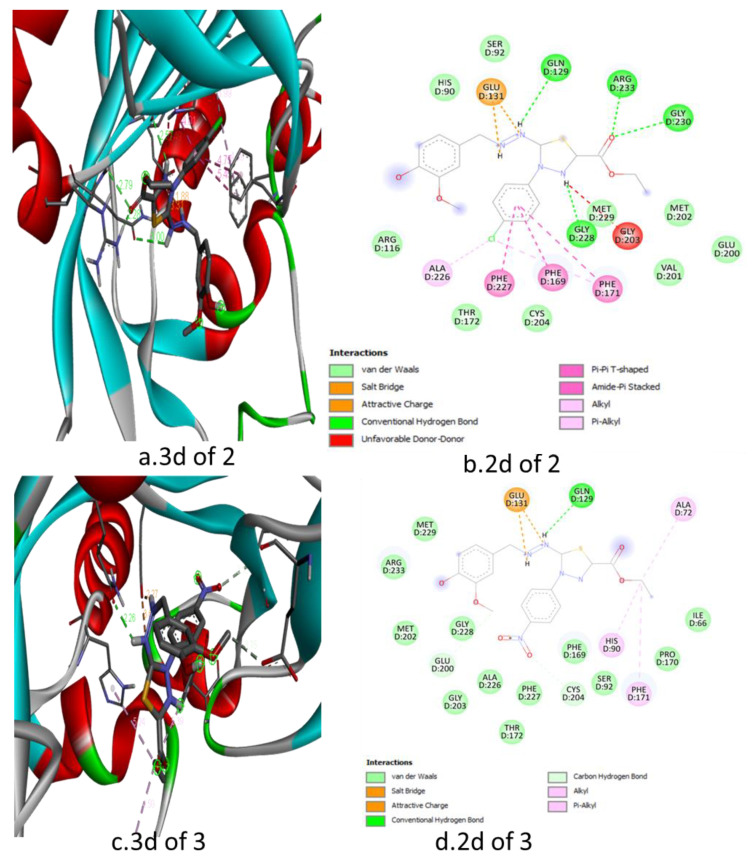
Interaction of the docked compounds **2** and **3** with PheRS.

**Figure 4:**
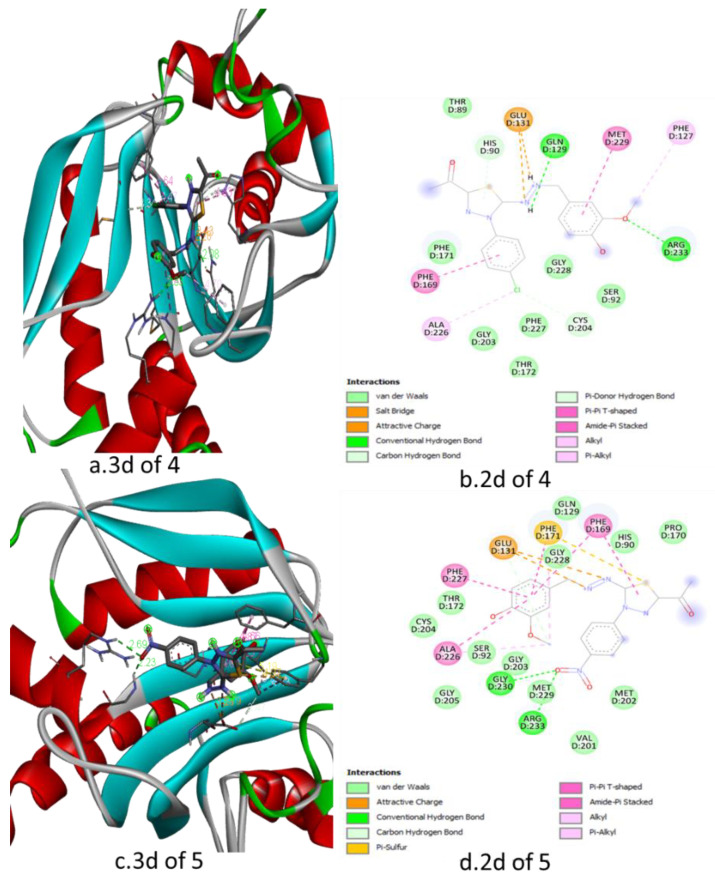
Interaction of the docked compounds **4** and **5** with PheRS.

**Figure 5:**
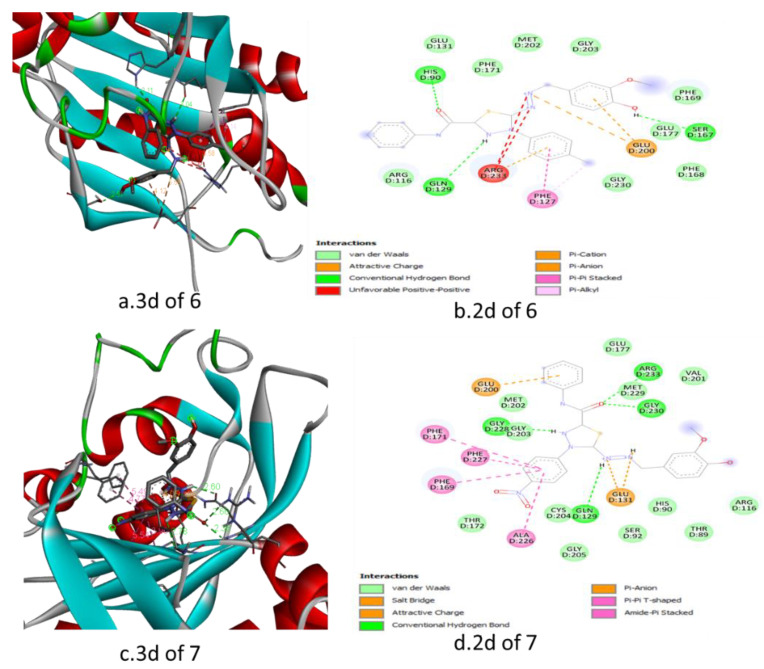
Interaction of the docked compounds **6** and **7** with PheRS.

**Figure 6:**
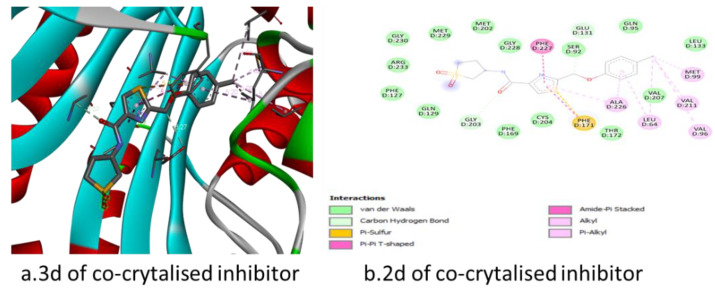
Interaction of the docked co-crystallized substrate with PheRS.

Phenylalanyl (Phe)-tRNA synthetase (PheRS) is an essential enzyme that catalyzes the transfer of phenylalanine to the Phe-specific transfer RNA (tRNA(Phe)), an important step in protein biosynthesis [52]. This enzyme is considered a druggable target for thiazole derivatives to develop antimicrobial drugs [53]. In the current study, compounds that could inhibit *Gram-negative* bacteria in vitro, could also bind to the pocket of PheRS in silico in a manner similar to its co-crystalized inhibitor (formed interactions with phe227, phe171, and ala226) as in Table 3 and Figure 3, Figure 4, Figure 5 and Figure 6. They could also excel over it by developing H-bonds in the pocket, which varied in position (Arg233, gly228, gly230, gln129), number, and length (from 2.08–3.04 Å). In vitro, compound **7** showed the highest inhibition compared to the others. It did the same in silico by forming the highest number of interactions (four H-bonds and other pi interactions) that qualified it to serve as a potential good inhibitor.

**Figure 7:**
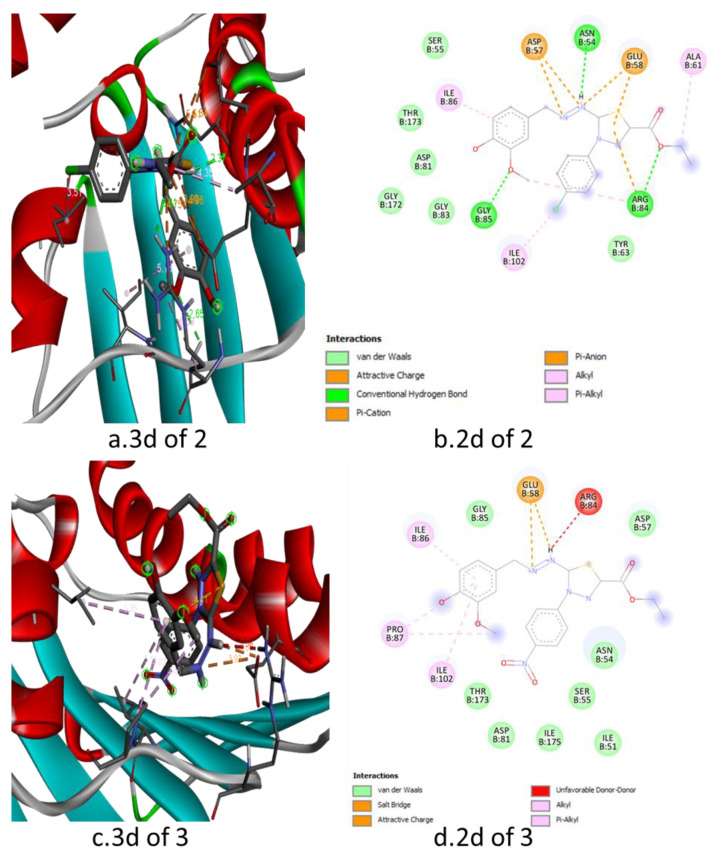
Interaction of the docked compounds **2** and **3** with bacterial DNA gyrase B.

**Figure 8:**
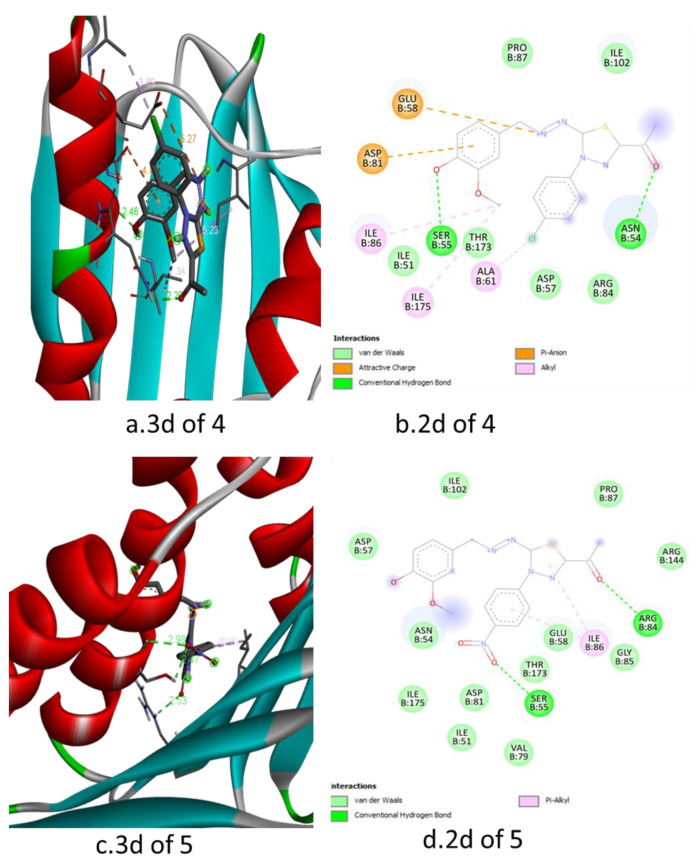
Interaction of the docked compounds **4** and **5** with bacterial DNA gyrase B.

**Figure 9:**
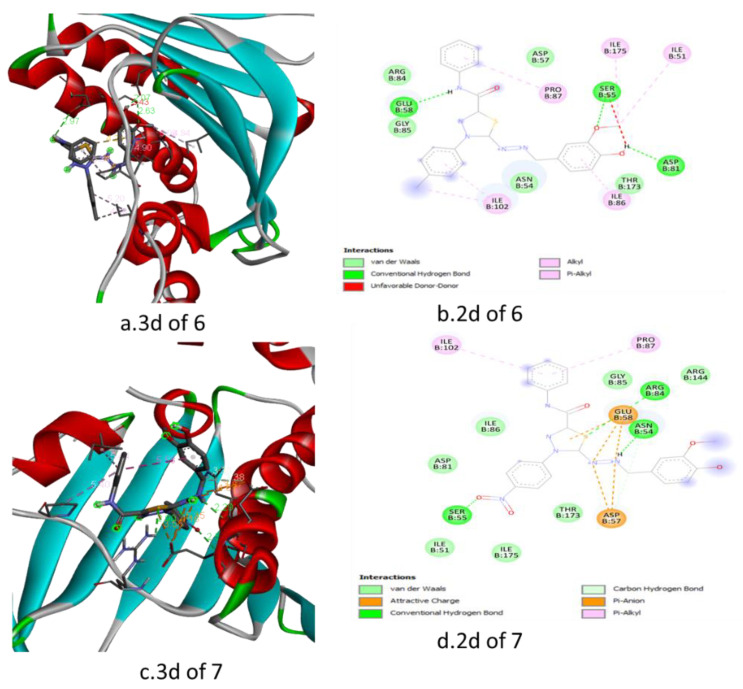
Interaction of the docked compounds **6** and **7** with bacterial DNA gyrase B.

**Figure 10:**
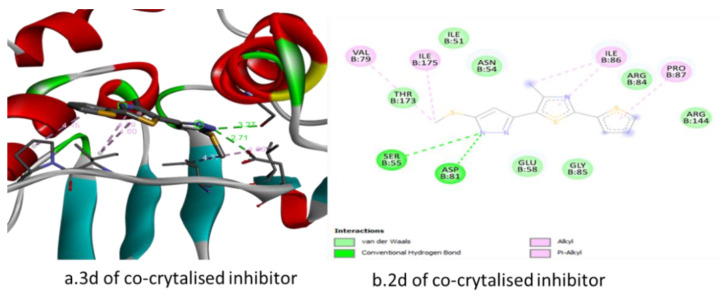
Interaction of the docked co-crystalized substrate with bacterial DNA gyrase B.

Bacterial DNA gyrase B (GyrB), an enzyme that negatively double-stranded DNA, became an attractive target for the examination of new antibacterial agents. Thiazoles were identified as having an inhibitory effect on the GyrB, with its ATP-binding site [54]. As represented in Table 4 and Figure 7, Figure 8, Figure 9 and Figure 10, the in silico results confirmed the in vitro results where compounds **2**, **4**, **5**, **6**, **7** showed good inhibitory effects on Gram-positive bacteria. Similar to the co-crystalized inhibitor, they could interact with residues such as ile175, ile86, and pro87, including the H-bonds, forming resides, ser55 and asp81. Compounds **2** and **7** formed a higher number of H-bonds (3). The compounds showed better binding compared to the co-crystalized inhibitor of GyrB in terms of binding energy, nature, number, and types of the formed bonds/ interactions.

**Figure 11:**
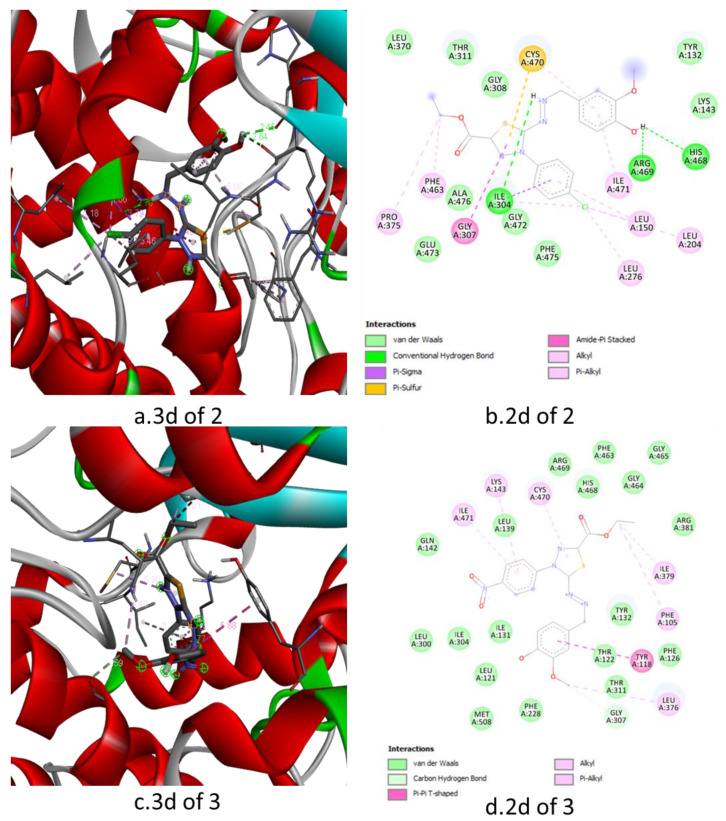
Interaction of the docked compounds **2** and **3** with fungal sterol 14α-demethylase (CYP51).

**Figure 12:**
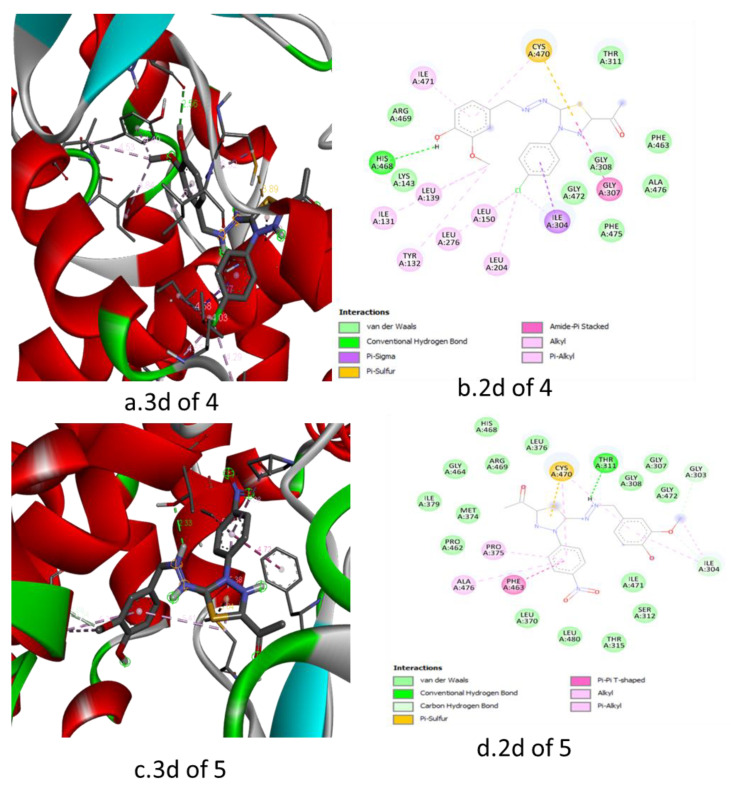
Interaction of the docked compounds **4** and **5** with fungal sterol 14α-demethylase (CYP51).

**Figure 13:**
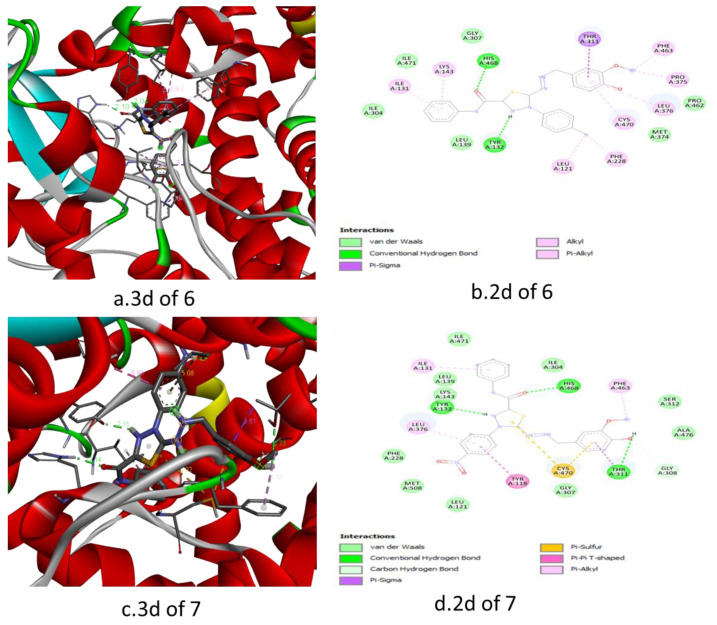
Interaction of the docked compounds **6** and **7** with fungal sterol 14α-demethylase (CYP51).

**Figure 14:**
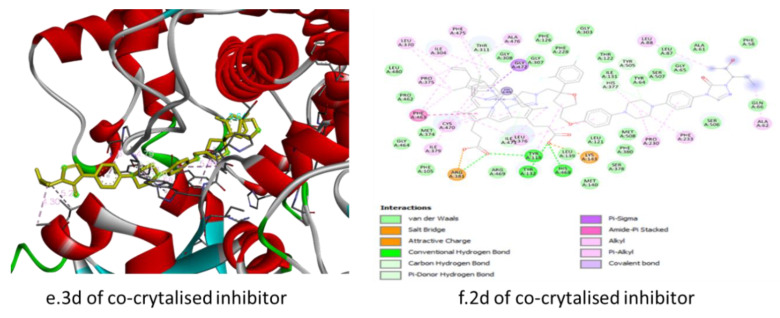
Interaction of the docked compounds **6** and **7,** and the co-crystalized substrate with fungal sterol 14α-demethylase (CYP51).

Sterol 14α-demethylase (CYP51) is an essential cytochrome P450 enzyme for sterols biosynthesis in fungi and is a key target of clinical drugs for controlling fungal diseases [55]. In the current study, among the experimentally tested compounds, only compounds **2**, **4**, **5**, **6**, and **7** exhibited a promising antifungal activity on the tested fungal strain at roughly close strengths with better advantage for compound **7** over the others. This could be interpreted in the light of the docking results where compounds could form interactions within the pocket of CYP51 as with those developed by the standard inhibitor posaconazole (residues leu376 and tyr118) as shown in Table 5 and Figure 11, Figure 12, Figure 13 and Figure 14. The new compounds could also form new hydrogen bonds, e.g., ile304, Thours311, his468, tyr132, arg469, which stabilize the compounds in the pocket and make them good inhibitors of CYP51. This represents a good step toward developing new antimicrobial agents for any future antifungal resistance that might emerge for posaconazole, where the compounds formed new interactions different from posaconazole.

In the light of in vitro and silico results, one could recommend compounds **2**, **6**, and **7** for further steps of drug development as antimicrobial agents.

## 4. Experimental Section

### 4.1. Chemistry

#### 4.1.1. Experimental Instrumentation

All melting points were determined on an electrothermal apparatus and are uncorrected. IR spectra were recorded (KBr discs) on a Shimadzu FT-IR 8201 PC spectrophotometer. ^1^H- and ^13^C-NMR spectra were recorded in (CD3)_2_SO solutions on a BRUKER 500 FT-NMR system spectrometer, and chemical shifts are expressed in ppm units using TMS as an internal reference. Mass spectra were recorded on a GC-MS QP1000 EX Shimadzu. Elemental analyses were carried out at the Microanalytical Center of Cairo University, Cairo, Egypt.

#### 4.1.2. Synthesis of Compounds

##### General Procedures for Synthesis of Molecules **2**–**7**

Methyl-2-(4-hydroxy-3-methoxybenzylidene)hydrazine-1-carbodithioate (**1**) acted as key molecule to produce new 1,3,4-thiadiazole derivatives by its reaction with the appropriate hydrazonoyl halides utilizing the grindstone chemistry; in detail, a mixture of 1.28 mg (5 mmol) of compound **1** and the appropriate hydrazonoyl halides (5 mmol) were ground with a pestle in an open mortar at RT with the addition of few drops (2–3) of DIPEA (diisopropyl ethyl amine) as catalytic amount, for 3–5 min till the mixture turned into the melt. The initial syrup grinding continued for 5–15 min, and the reaction was monitored by TLC (Thin Layer Chromatography) using ethyl acetate/petroleum ether (1:2, *v*/*v*) as eluent. The solid was collected, washed with water, and then ethanol and recrystallized from the proper solvent to afford the desired molecules **2**–**7**.

##### Ethyl-4-(4-chlorophenyl)-5-(-4-hydroxy-3-methoxybenzylidene)hydrazono)-4,5-dihydro-1,3,4-thiadiazole-2-carboxylate (**2**)

Yellow solid (75%); m.p. 161–163 °C, FT-IR (KBr, cm^−1^): *v* 3481 (OH), 1725 (C=O), 1599 (C=N), 1554 (C=C); ^1^H-NMR (DMSO-d*_6_*): δ 1.27 (t, 3H, CH_2_**CH_3_**), 3.72 (s, 3H, O**CH_3_**), 4.12 (q, 2H, **CH_2_**CH_3_), 7.33–7.45 (m, 7H, ArH), 8.29 (s, 1H, CH), 9.65 (s, 1H, OH);^13^C-NMR (100 MHz, DMSO-d*_6_*): δ 13.57 (CH_3_), 55.52 (OCH_3_), 63.72 (CH_2_), 111.18 (Ar), 117.60 (Ar), 122.51 (Ar), 123.55 (Ar), 124.50 (Ar), 127.31(Ar), 128.90 (Ar), 129.28 (Ar), 137.62 (Ar), 142.27(Ar), 146.92 (Ar), 149.52 (CH), 156.12 (Ar), 157.06 (C=N), 161.72(C=O); MS *m/z* (%): 434 (M + 2, 27), 432 (M^+^, 25). Anal. Calcd. for C_19_H_17_ClN_4_O_4_S (432): C, 52.72; H, 3.96; N, 12.94. Found: C, 52.65; H, 3.92; N, 12.89%.

##### Ethyl-5-((-4-hydroxy-3-methoxybenzylidene)hydrazono)-4-(4-nitrophenyl)-4,5-dihydro-1,3,4-thiadiazole-2-carboxylate (**3**)

Yellow solid (82%); m.p. 182–184 °C, FT-IR: *v* 3522 (OH), 1715 (C=O, carbonyl ester), 16,050 (C=N), 1565 (C=N); ^1^H-NMR: δ 1.22 (t, 3H, CH_2_**CH_3_**), 3.85 (s, 3H, OCH_3_), 4.42 (q, 2H, **CH_2_**CH_3_), 7.31–7.33 (m, 7H, ArH), 8.22 (s, 1H, CH), 9.75 (s, 1H, OH); ^13^C-NMR: δ 13.97 (CH_3_), 54.53 (OCH_3)_, 62.71 (CH_2_), 111.19 (Ar), 115.58 (Ar), 121.37 (Ar), 123.52 (Ar), 126.51 (Ar), 128.47 (Ar), 135.19 (Ar), 136.90 (Ar), 142.90 (Ar), 147.93 (Ar), 149.55 (CH), 151.93 (Ar), 157.07 (C=N), 162.62 (C=O); MS *m/z* (%): 443 (M^+^, 27). Anal. Calcd. for C_19_H_17_N_5_O_6_S (443): C, 51.46; H, 3.86; N, 15.79. Found: C, 51.42; H, 3.92; N, 15.85%.

##### 1-(4-(4-Chlorophenyl)-5-(4-hydroxy-3-methoxybenzylidene)hydrazono)-4,5-dihydro-1,3,4-thiadiazol-2-yl)ethan-1-one (**4**)

Orange crystals from acetic acid (72%); m.p. 214–216 °C, FT-IR: *v* 3495 (OH), 1678 (C=O), 1600 (C=N), 1557 (C=C); ^1^H-NMR: δ 2.47 (s, 3H, CH_3_), 3.65 (s, 3H, OCH_3_), 7.54–7.85 (m, 8H, ArH), 8.22 (s, 1H, CH), 9.55 (s, 1H, OH); ^13^C-NMR: δ 25.13 (CH_3_), 55.53 (OCH_3_), 111.02 (Ar), 115.60 (Ar), 122.60 (Ar), 124.49 (Ar), 125.12 (Ar), 127.14 (Ar), 137.53 (Ar), 145.19 (Ar), 149.55 (CH), 151.16 (Ar), 155.37 (C=N), 165.03 (C=O); MS *m/z* (%): 404 (M + 2, 20), 402 (M^+^, 18); Anal. Calcd. for C_18_H_15_ClN_4_O_3_S (402): C, 53.67; H, 3.75; N, 13.91. Found: C, 53.62; H, 3.71; N, 13.89%.

##### 1-(5-(4-Hydroxy-3-methoxybenzylidene)hydrazono)-4-(4-nitrophenyl)-4,5-dihydro-1,3,4-thiadiazol-2-yl)ethan-1-one (**5**)

Orange crystals from acetic acid (61%); m.p. 191–193 °C, FT-IR: *v* 3502 (OH), 1681 (C=O), 1600 (C=N); ^1^H-NMR: δ 2.51 (s, 3H, CH_3_), 3.79 (s, 3H, OCH_3_), 7.30–7.32 (m, 7H, ArH), 8.27 (s, 1H, CH), 9.54 (s, 1H, OH); ^13^C-NMR: δ 20.65 (CH_3_), 57.51 (OCH_3_), 110.92 (Ar), 111.52 (Ar), 123.56 (Ar), 125.51 (Ar), 127.50 (Ar), 136.27 (Ar), 137.57 (Ar), 145.82 (Ar), 147.53 (Ar), 149.12 (CH), 156.28 (C=N), 164.12 (C=O); MS *m/z* (%): 413 (M^+^, 15)%. Anal. Calcd. for C_18_H_15_N_5_O_5_S (413): C, 52.30; H, 3.66; N, 16.94. Found: C, 52.28; H, 3.57; N, 16.98%.

##### 5-((-4-Hydroxy-3-methoxybenzylidene)hydrazono)-*N*-phenyl-4-(*p*-tolyl)-4,5-dihydro-1,3,4-thiadiazole-2-carboxamide (**6**)

Yellow crystals from ethanol, m.p. 222–224 °C; yield (85%); FT-IR: *v* 3451 (OH), 3387, 1681 (C=O), 1578 (C=C); ^1^H-NMR: δ 2.57 (s, 3H, CH_3_), 3.73 (s, 3H, OCH_3_), 6.86–7.72 (m, 12H, ArH), 8.38 (s, 1H, CH), 9.85 (s,1H, OH), 10.72 (s, 1H, NH); ^13^C-NMR: δ 20.21(CH_3_), 56.07 (OCH_3_), 112.05 (Ar), 115.61 (Ar), 122.06 (Ar), 123.14 (Ar), 124.20 (Ar), 125.50 (Ar), 127.14 (Ar), 128.15 (Ar), 134.54 (Ar), 135.12 (Ar), 148.13 (CH), 151.04 (Ar), 154.89 (C=N), 164.16 (C=O); MS *m/z* [%]: 445 (M^+^), 459 (75); Anal. Calcd. for C_24_H_21_N_5_O_3_S (459): C, 62.73; H, 4.61; N, 15.24%. Found: C, 62.78; H, 4.58; N, 15.21%.

##### 5-((-4-Hydroxy-3-methoxybenzylidene)hydrazono)-4-(4-nitrophenyl)-*N*-phenyl-4,5-dihydro-1,3,4-thiadiazole-2-carboxamide (**7**)

Yellow crystals from ethanol, m.p. 182–184 °C; yield (75%); FT-IR: *v* 3447 (broad band, NH, OH), 1661 (C=O), 1600 (C=N), 1539 (C=C); ^1^H-NMR: δ 3.52 (s, 3H, OCH_3_), 7.22–7.95 (m, 12H, ArH), 8.36 (s, 1H, CH), 9.65 (s,1H, OH), 10.58 (s, 1H, NH); ^13^C-NMR: δ 52.5 (OCH_3_), 110.15 (Ar), 118.81 (Ar), 122.76 (Ar), 122.43 (Ar), 123.77 (Ar), 124.58 (Ar), 127.94 (Ar), 128.22 (Ar), 135.14 (Ar), 136.24 (Ar), 145.33 (Ar), 149.15 (CH), 155.89 (Ar), 156.26 (C=N), 162.16 (C=O); MS *m/z* [%]: 491 (M + 1, 10), 444 (75), 370 (32), 281 (18), 225 (81), 155 (12), 127(18), 66 (25); Anal. Calcd. for C_23_H_18_N_6_O_5_S (490): C, 56.32; H, 3.70; N, 17.13%. Found: C, 56.27; H, 3.65; N, 17.09%.

### 4.2. Biological Activities

Two strategies were employed in the antibacterial and antifungal assessment. The first was the Kirby–Bauer disc diffusion method, which was utilized to determine the width of the zone of inhibition (ZOI) for each bacterial and fungal strain. The second strategy, microbial growth inhibition, was utilized to define the minimum inhibitory concentration (MICs) values of synthesized compounds on bacterial and fungal strain proliferation.

#### 4.2.1. Preparation of Stock Solution

A standard stock solution of each compound was assembled by dissolving 10 mg of each compound in 1 mL of 10% dimethyl sulfoxide (DMSO) as a solvent [56].

#### 4.2.2. Microorganisms Used

The susceptibility of four distinct microbial species to synthetic compounds was examined. In this research, *Rhizopus oryzae* was applied as a model for fungal strain. Likewise, four bacterial species were used, namely *Pseudomonas aeruginosa* and *Klebsiella pneumoniae* as a model for Gram-negative species, and *Staphylococcus aureus* and *Bacillus subtilis* as a model for Gram-positive strains. The conserved bacterial and fungal cultures were re-cultivated by transferring a single colony of each organism into tubes containing 10 mL of nutrient broth for bacteria and Sabouraud Dextrose broth for fungi, and all inculcated tubes were incubated for 18–24 h at 37 °C for bacteria and 3–4 days at 28 °C for fungi [57].

#### 4.2.3. Kirby–Bauer Disc Diffusion Method

Disc diffusion assay was utilized to scrutinize the antibacterial and antifungal potential of the synthesized compounds versus fungal and bacterial species. In a Petri dish (60 mm), 15–20 mL agar of Sabouraud Dextrose agar (SDA) for fungal species and Mueller–Hinton agar (MHA) for bacterial species was deposited. Afterward, broth cultures adjusted to 0.5 McFarland turbidity (10^8^ CFU/mL) were implanted onto the agar surface. On the top of the plate inoculated with a standardized suspension of microbes to be investigated, discs containing known amounts (50 µL) of the studied compound were inserted. Negative controls included paper discs containing solely dimethyl sulfoxide (DMSO). Positive controls were ciprofloxacin (30 µg) (Sigma-Aldrich, Steinheim, Germany) for bacteria and amphotericin B (10 µg) (Sigma-Aldrich, Steinheim, Germany) for fungi. A solvent-only control test was also carried out. The MHA and SDA plates were incubated at the optimal conditions for each microorganism; the thickness of the zone of inhibition (ZOI) formed in surrounding discs was measured (mm). All experiments were accomplished in triplicate, and if the results were different, they were repeated [58].

#### 4.2.4. Determination of Minimum Inhibitory Concentration (MIC)

Minimum inhibitory concentrations (MICs) were employed to explore antimicrobial activity. The broth dilution technique was operated to estimate the MIC of each compound. A volume of stock solution was prepared formerly of each test compound, and was serially diluted to reach concentrations ranging from 50 to 300 µg/mL for antibacterial potential, while 4–32 mg/mL for antifungal activities. Each concentration of the studied compound was then added to culture media in a test tube at varying concentrations, and different strains were injected at a concentration of 10^8^ CFU/mL. Culture media included nutrition agar (for antibacterial) and Sabouraud dextrose agar medium (for antifungal). All tested tubes were incubated at 37 °C (antibacterial) or 30 °C (antifungal) for 3–4 days before being checked for the presence or absence of the tested microbial growth. The MIC values were calculated using the lowest concentration of test compounds at which the tubes stayed clean, signifying that bacterial or fungal proliferation was suppressed entirely [59].

#### 4.2.5. Minimum Biocidal Concentrations (MBCs)

In total, 10 µL aliquots from each test tube that exhibited no microbial proliferation were streaked on MHA and SDA plates and incubated at 37 °C for 24 h (bacteria) and 72 h (fungi) to determine the MBC values.

#### 4.2.6. ATP Bioluminescence Assay

Extracellular ATP levels, which indicate activities and vitals in all microbial cells, were estimated using the luciferin-luciferase process. In total, 30 μL ATP aliquot suspension was mixed with 270 μL luciferin-luciferase aliquot mixture during this direct assay. Finally, the mixed suspension was allocated for spectroscopic analysis as an optical detection system. Using the ATP luminometer, luminescence patterns and luminescence intensity were assessed and expressed as relative illumination units (RLU) [60,61]

#### 4.2.7. Toxicological Performance Assay

Microtox^®^ Model 500 Analyzer (Modern Water, New Castle, DE, USA) was applied to measure each compound’s toxicity level and EC_50%_. The toxic influences of all studied compounds were ascertained to guarantee their safe and fruitful benefit for pharmaceutical and biomedical purposes without any unfavorable impacts on individuals. The toxicity grade was reckoned at the highest concentration (300 µg/mL) for each studied compound [61].

The cytotoxicity assay on the HEp-2 cell lines by colorimetric MTT assay was applied to determine the percentage of surviving cells. MTT (3,4,5-(dimethylthiazol-2-yl) 2-5-diphenyl tetrazolium bromide) is reduced to its violet formazan product by metabolically active cells; the MTT assay was utilized to determine the compound’s cytotoxicity. As indicated above, the cells were subjected to multiple compound concentrations and kept in the incubator for 24 h at 37 °C (Soysa et al. 2014) [62].

## 5. Molecular Docking

The X-ray crystal structure of bacterial phenylalanyl-tRNA synthetase (PheRS) complex with its co-crystallized inhibitor (*N*-[(3*S*)-1,1-dioxidotetrahydrothiophen-3-yl]-2-[(4-methylphenoxy) methyl]-1,3-thiazole-4-carboxamide), DNA Gyrase B (GyrB) complexed with its co-crystallized inhibitor (4-methyl-5-[3-(methylsulfanyl)-1*H*-pyrazol-5-yl]-2-thiophen-2-yl-1,3-thiazole) and the fungal sterol 14α-demethylase (CYP51) with its co-crystallized inhibitor (posconazol) were obtained from protein databank using http://www.rcsb.org, accessed on 8 May 2022 (PDB id: 4P74, 3G75 and 5FSA respectively) to be docked against the tested compounds. The protein structure was prepared for docking by eliminating water molecules and the co-crystalized ligand (androstenedione). Hydrogens were added then protein structure was saved as PDB file by Biovia Discovery studio 2021. The compounds from **2** to **7** were docked against “4P74, 3G75 and 5FSA” proteins, with cavity space dimensions (x: 43.3119, y: −21.1489, z: 22.0329), (x: 7.4930, y: −8.4470, z: 1.2665), and (x: 193.4472, y: −2.1514, z: 38.0304), respectively. Docking was performed using Autodock Vina’s scoring function of the graphic user interface software PyRx 0.8.

## 6. Conclusions

The current investigation has potential biomedical implications as the antibacterial and antifungal properties of specific synthetic compounds were successfully evaluated. This serves as a foundation for ongoing studies into alternative antibiotics which are hoped to develop more promising therapies against potential pathogens, especially black fungus and pathogenic bacteria. The results show that compound **7** has remarkable antimicrobial features and potential for pharmaceutical use as a new medication candidate against bacterial and fungal infections. Some of the studied compounds, including compounds **6** and **7**, have been ascertained to be promising nominees for additional effectiveness evaluation based on the attained results. Moreover, the computational study revealed that compounds **2**, **6**, and **7** showed the best interactions with the selected protein targets.

## Figures and Tables

**Table 1:** Antimicrobial properties and ZOI diameters of six newly synthesized compounds against particular pathogenic microbes.

Compounds	Diameters of ZOI (mm)				
	Gram-Negative Species		Gram-Positive Species		Fungal Strain
	*K. pneumoniae*	*P. aeruginosa*	*S. aureus*	*B. subtilis*	*R. oryzae*
**2**	19 ± 0.10	20 ± 0.23	13 ± 0.18	15 ± 0.25	9.7 ± 0.11
**3**	13 ± 0.23	15 ± 0.36	10 ± 0.18	12 ± 0.29	10 ± 0.14
**4**	16 ± 0.18	18 ± 0.28	13 ± 0.25	15 ± 0.42	12 ± 0.10
**5**	15 ± 0.35	17 ± 0.24	12 ± 0.30	14 ± 0.18	11 ± 0.14
**6**	20 ± 0.18	22 ± 0.23	15 ± 0.26	17 ± 0.16	14 ± 0.28
**7**	22 ± 0.16	25 ± 0.14	18 ± 0.28	20 ± 0.43	17 ± 0.14
**Cip.**	18 ± 0.35	20 ± 0.12	15 ± 0.43	17 ± 0.29	nd
**Amp.**	nd	nd	nd	nd	21 ± 0.15

**Cip.** = ciprofloxacin, **Amp.** = amphotericin B, nd = not determined.

**Table 2:** Minimum inhibitory concentrations (MICs) and minimum bactericidal concentrations (MBCs) of studied newly synthesized compounds against microbial strains.

Compounds	Assays (µg/mL)	Gram-Negative Species		Gram-Positive Species		Fungal Strain
		*K. pneumoniae*	*P. aeruginosa*	*S. aureus*	*B. subtilis*	*R. oryzae*
**2**	MIC	175	125	200	175	225
	MBC	225	175	225	200	250
**3**	MIC	275	250	300	275	>300
	MBC	300	275	>300	>300	>300
**4**	MIC	225	200	250	225	275
	MBC	275	250	>300	>300	>300
**5**	MIC	225	200	250	225	275
	MBC	275	250	>300	>300	>300
**6**	MIC	150	125	175	150	225
	MBC	200	175	225	200	275
**7**	MIC	75	75	125	100	150
	MBC	125	100	175	150	200

**Table 3:** Interactions of the docked compounds with bacterial PheRS.

Compound	Binding Energy (kcal/mol)	H-Bonds	Residual Interactions
**2**	−8.2	(4): Arg233, gly228, gly230, gln129	phe227, glu131, phe171, phe169, ala226,
**3**	−8.4	(1): gln129	glu131, ala72, his90, phe171, cys204, glu200
**4**	−8.1	(2): Arg233, gln129	cys204, phe169, ala226, met229, phe127
**5**	−8.4	(2): Arg233, gly230	phe227, glu131, phe171, phe169, ala226,
**6**	−9.2	(3): gln129, his90, ser167	phe227, glu200, arg233
**7**	−9.0	(4): Arg233, gly228, gly230, gln129	phe227, phe221, phe171, phe169, ala226, glu131, glu200
Co-crystalized inhibitor	−9.8	(Zero)	phe227, phe171, val211, ala226, val207, gly203, met99, leu64, val96

**Table 4:** Interactions of the docked compounds with bacterial DNA gyrase B.

Compound	Binding Energy (kcal/mol)	H-Bonds	Residual Interactions
**2**	−7.2	(3): Arg84, gly85, asn54	Glu58, asp57, ile102, ile86, ala61
**3**	−6.8	(zero)	Glu58, ile102, ile86, pro87
**4**	−6.6	(2): ser55, asn54	asp81, Glu58, ile175, ile86, ala61
**5**	−6.9	(2): Arg84, ser55	ile86
**6**	−7.5	(3): ser55, asp81, Glu58	ile175, ile51, pro87, ile102, ile86
**7**	−7.3	(3): Arg84, ser55, asn54	Glu58, asp57, ile102, pro87
Co-crystalized inhibitor	−6.4	(2): ser55, asp81	Val79, ile175, ile86, pro87

**Table 5:** Docking results of the new compounds with sterol 14α-demethylase (CYP51).

Entry	Binding Energy (kcal/mol)	H-Bonds		Residual Interactions
		Number	Residues	
**2**	−8.3	3	ile304, his468, arg469	Leu276, phe463, leu204, pro375, cys470, ile471, gly307, leu150, ile304, ile471
**3**	−8.3	0	NA	leu376, ile379, Phe105, tyr118, gly307, ile471, lys143
**4**	−8.3	1	his468	Leu204, ile304, cys470, gly307, leu276, ile131, ile471, tyr132
**5**	−8.8	1	Thours311	Ala476, cys470, pro375, gly303, phe463, ile304
**6**	−9.4	2	his468, tyr132	Thours311, lys143, phe228, pro375, phe463, leu376, leu121, ile131, cys470
**7**	−10.1	3	Thours311, his468, tyr132	phe463, leu376, leu131, cys470, gly308, tyr118
Co-crystalized inhibitor	−12.2	0	NA	Ala62, try505, leu88, phe233, pro230, leu376, tyr118, his377

## Data Availability

Not applicable.

## Sample Availability

Samples of the compounds are not available from the authors.
